# Goodwill Towards Men

**Published:** 1889-12-21

**Authors:** 


### Words of Consolation.

### GOODWILL TOWARDS MEN.

*(For Reading to the Sick.)*

At this season of the year it seems specially hard that any of us should be laid on the bed of sickness, and deprived of that festivity and feasting which celebrate the birthday of our Saviour. When family parties gather in the beloved old home, how hard it is for one to be left in the long, bare ward, alone amongst strangers. It indeed needs Christian resignation to accept this trial with cheerfulness, and hence it is specially necessary to recall to the sick their duty to show goodwill towards men.

Christmas morning in the hospital! Well, the thought may cause a shudder, but a little unselfishness all round, an effort on the part of each to induce peace and goodwill, and the reality will not be so dreadful after all. It is one of the blessings of the spiritual life that it is "hid in Christ with God"; that loneliness and pain and trials of earth can never penetrate to its peaceful depths. Alone on a sick-bed with pain-racked limbs the body may be—but the mind can fly to its heavenly refuge and revel in sweet thoughts and communion with the saints.

But the inward life is only rightly used as a period of refreshment to help us with our outward life. We are now on the earth; now is the time to fight and conquer, to prove of what sort of stuff we are made, and whether we are worthy of the life to come. So at Christmas time we are bound to try and further earthly peace and to work goodwill amongst our neighbours. Even though sick unto death, our duty is still towards those around us: a word of kindness to the patient in the next bed, a cheerful word of greeting to doctor or nurse, a little self-control to still our groans and hide our pains. And those who have greatly suffered, who know what pangs poor humanity has sometimes to bear, are those who are best able to show by their courage and patience that peace and goodwill can endure through all. No matter how the head aches or the fever burns, during this Christmas season let us only think of our blessings and our mercies, and strive through all to be a help to those who keep festival, and not a drawback to the universal rejoicing. No man is so poor or so ill but he can forward the cause of goodwill and peace; and if the reason for our festival be remembered, surely it is easy to find cause for joyfulness. The martyrs of old sang hymns of praise while the fire was burning their limbs; the young virgins walked towards the lions, seeing them not, their faces beaming with heavenly light as they gazed upwards towards their God. The thought of Christ so filled them they felt no pain, or, at least, were able to conquer pain and glory in their sufferings. Some of this holy ecstasy might mitigate our griefs of to-day if we would only dwell on the love of our Saviour and not on our own trials and troubles. Then, full of cheerful resignation and forgetfulness of self, we can join in the hymn:

Hail the heaven-born Prince of Peace,
Hail the Sun of Righteousness!
Light and life to all He brings
Risen with healing in His wings.

